# A Case Report of Severe Hyponatremia Secondary to Coronavirus Disease 2019 Viral Pneumonia

**DOI:** 10.7759/cureus.14077

**Published:** 2021-03-24

**Authors:** Vinati Pillutla, Anooj Patel, Sindhoora Koneru, Kenneth K Ng, Mitchell K Ng

**Affiliations:** 1 Internal Medicine, Indus Hospital, Visakhapatnam, IND; 2 Plastic Surgery, Case Western Reserve University School of Medicine, Cleveland, USA; 3 Department of Biomedical Sciences, Texas A&M University, College Station, USA; 4 Anesthesiology, State University of New York Downstate Health Sciences University, Brooklyn, USA; 5 Orthopaedic Surgery, Maimonides Medical Center, Brooklyn, USA

**Keywords:** acute hyponatremia, coronavirus disease 2019 (covid-19), severe acute respiratory syndrome coronavirus-2 (sars-cov-2)

## Abstract

As of December 2020, the coronavirus disease 2019 (COVID-19) pandemic has resulted in 82.2 million cases worldwide. We report the case of a 69-year-old South Asian female with a history of hypertension, hypothyroidism, meningiomatosis, and urinary incontinence who contracted COVID-19 and developed severe hyponatremia. She was initially medically managed with antibiotics, anti-parasitics, anti-coagulants, and steroids. After experiencing breathlessness, chest discomfort, high systolic blood pressure, and tachycardia, she was admitted and diagnosed with post-COVID pneumonia, and was conservatively treated with steroids. She showed improvement, and was discharged upon being declared hemodynamically stable. While the patient was at home, she experienced periods of breathlessness and acral edema. This case raises the question of the correlation between hyponatremia and COVID-19, especially in regards to symptomatic presentations, including altered mental status, headache, and nausea. As there are limited studies that show severe electrolyte disorders leading to mortality, more research is needed to understand hyponatremia in cases with COVID-19.

## Introduction

By the end of December 2020, there were 82.2 million cases of coronavirus disease 2019 (COVID-19), 1.79 million confirmed deaths, and 58.3 million discharged or recovered cases worldwide [1]. It is well documented that mortality is higher in patients with significant comorbidities and those within older age groups. Mortality rate is estimated at 31.8% in patients aged 85 and older and 59.1% in patients aged 75 and older. Patients with preexisting conditions, usually cardiovascular (20.8%) and chronic respiratory (35.9%) diseases, accounted for higher fatalities compared to those with no prior health conditions [2]. Though the most common cause of COVID-19 is severe acute respiratory syndrome coronavirus-2, few cases are being reported where patients exhibit severe hyponatremia associated with COVID-19 [3,4]. While there is not enough data to establish a direct correlation between hyponatremia and COVID-19, there are several reported cases of patients with COVID-19 exhibiting low plasma sodium levels [5].

Here, we review the case of a 69-year-old female, who developed severe hyponatremia after contracting COVID-19, with no prior history of electrolyte abnormalities. This case highlights a possible association between hyponatremia and COVID-19, especially in regards to symptomatic presentation, including headache, malaise, nausea, and altered mental status.

## Case presentation

**Figure 1:**
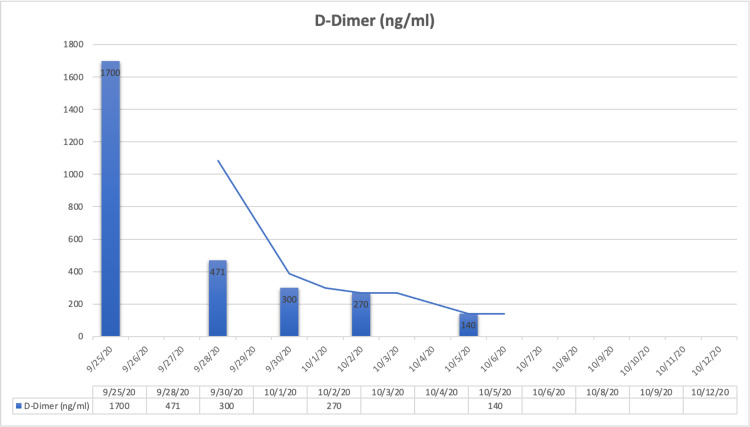
Trend of patient’s D-dimer levels throughout hospital admission.

We present the case of a 69-year-old South Asian female with a past medical history of high blood pressure, hypertension, hypothyroidism, meningiomas, and urinary incontinence. Her reverse-transcriptase polymerase chain reaction test was positive for COVID-19, and she was advised home isolation. Though she initially responded to the medication including antibiotics (doxycycline), anti-parasitic (ivermectin), anti-coagulant (apixaban), and adrenocortical steroids (Medrol), she was admitted into emergency with complaints of chest discomfort, breathlessness, tachycardia (average of 156 for a two-hour period), and hypertension (systolic pressure was 180 mmHg) four weeks later. On evaluation, she was found to have high D-dimer (1,700 ng/mL when the biological reference range is 0-400 ng/mL) (Figure 1) and residual lung lesions on computed tomography (CT) of the chest, which are typical for COVID-19 patients. Her CT pulmonary angiogram was negative with no evidence of pulmonary thrombo-embolism. The main, right, and left pulmonary arteries appeared normal.

Her breathlessness exacerbated with hypertension and she was considered for ventilation. She was given metoprolol to address her hypertension and better control her heart rate, along with low-molecular-weight heparin and other supportive medications.

**Figure 2:**
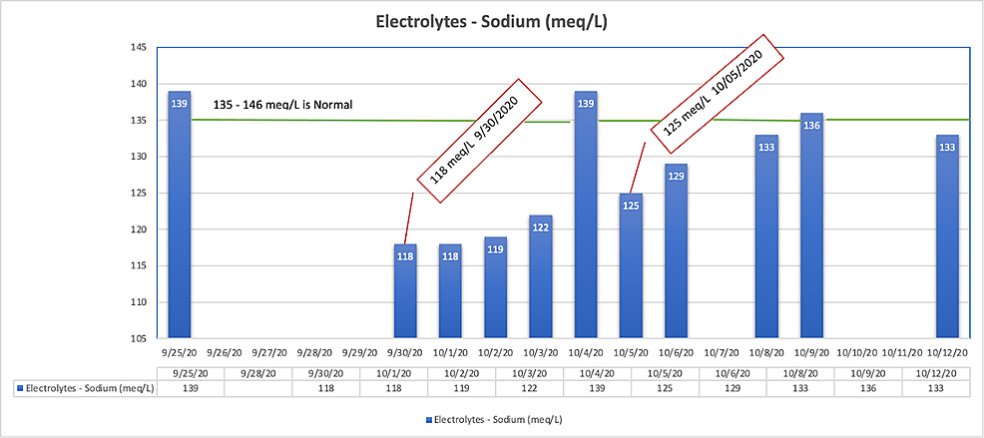
Trend of patient’s sodium levels during hospital admission.

A week into hospitalization, the patient exhibited severe levels of hyponatremia (118 mEq/L when the biological reference range is 135-146 mEq/L) (Figure 2) with symptoms including nausea, fatigue, and headache. She was treated with intravenous (IV) 3% saline followed by tolvaptan. Furthermore, during the course of hospitalization, she developed intermittent low-grade fever, and her repeated C-reactive protein (CRP) level tests were normal. After responding well to oral moxifloxacin, she was considered to be stable and was discharged.

**Figure 3:**
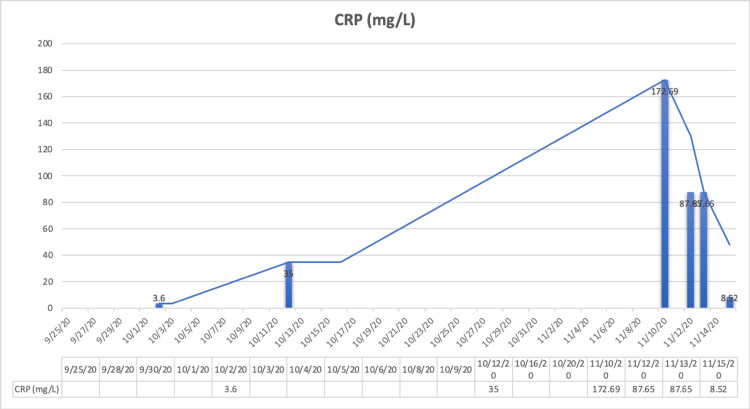
Trend of patient’s CRP levels during hospital admission. CRP, C-reactive protein

Three weeks later, she was admitted into the hospital again after experiencing nausea, fatigue, oral dryness, rashes, pain in hypochondriac areas, chest uneasiness, cold, cough, and persistent fever of 99-100 degrees Fahrenheit. She was advised to undergo CT chest, CRP, and serum electrolytes tests. Her CT chest showed multifocal patchy areas of ground glass opacities with associated septal thickening and interstitial bands in bilateral lower and upper lobes (with lower greater than upper) and lateral segment of right middle lobe suspicious for multifocal infectious pneumonitis, abnormalities typically found in COVID-19 patients. Her sodium levels were moderate at 129 mmol/L. Her CRP level was high (172.69 mg/L when the biological reference range in adults is <5 mg/L) (Figure 3). COVID rapid antigen test was done and was negative.

The patient was conservatively treated with steroids, antacids, antibiotics, and other supportive care. After showing significant improvement and decreased CRP levels, the patient was hemodynamically stable and was discharged.

## Discussion

This report presents a case of a 69-year-old female with no significant comorbidities, with the exception of hypothyroidism and high systolic blood pressure. Her condition deteriorated once she tested positive for COVID-19. She also exhibited severe hyponatremia with symptoms including nausea, headaches, and fatigue. While the cause of hyponatremia is often idiopathic, there are a host of well-described causes such as hormone imbalance [6], infections [7], heart failure, cirrhosis, syndrome of inappropriate antidiuretic hormone, and medications such as anticoagulants [8,9] that are potentially responsible. A correlation with COVID-19 is possible in this case as the patient did not have any history of low sodium levels. The patient never exhibited low sodium levels prior to COVID-19 infection. Hyponatremia in COVID-19 is poorly understood, and potential pathogenesis could involve antidiuretic hormone secretion in the context of elevated inflammatory cytokines such as interleukin-6 released by macrocytes and macrophages.

Hyponatremia is the most common electrolyte disorder in hospitalized patients and is defined as serum sodium concentration less than 135 mEq/L [10]. Hyponatremia can lead to serious morbidity and mortality [11] and presents in different forms [12]; hence, diagnosis and proper medical management play a critical role. The types of hyponatremia include hypervolemic, hypovolemic, and euvolemic hyponatremia. Given the patient’s clinical presentation and lack of hypervolemic (pitting edema of extremities, pulmonary edema) or hypovolemic symptoms (tachycardia, narrowed pulse pressure, low blood pressure), the patient most likely developed euvolemic hyponatremia. As stated earlier, she was given IV 3% saline and tolvaptan, both common ways to treat low sodium levels [10,13]. Our case also showed a W-curve [14] recovery in the patient as she was admitted to the hospital the second time in three weeks and was diagnosed as having post-COVID pneumonia and exhibited high CRP levels. The patient was treated with steroids, antacids, and antibiotics. She showed significant improvement, including reduced CRP levels. The patient was seen to be hemodynamically stable and was discharged. While hyponatremia is frequently associated with atypical pneumonia and seen in multiple cases [14], post-COVID pneumonia and acute hyponatremia in this patient leads us to explore an association of hyponatremia and COVID-19.

While the patient is at home with a long road to full recovery as she has periods of breathlessness and acral edema, this case highlights the need for further research to determine a potential correlation between hyponatremia and COVID-19. There are several cases of hyponatremia in conjunction with COVID-19, leading us to advise clinicians to check for hyponatremia in patients testing positive for COVID-19 [12]. It further emphasizes the need for clinicians to be vigilant and consider multi-organ and multi-system involvement in COVID-19 patients.

## Conclusions

This case highlights the occurrence of severe hyponatremia in a patient who had COVID-19 which ultimately led to pneumonia and a long road to recovery. The patient exhibited symptoms such as nausea, headaches, and fatigue, for which she was treated after being hospitalized. The first hospital visit was more intensive than the second as she had respiratory issues and was considered for intubation. We believe the patient exhibited euvolemic hyponatremia and the medications given to treat COVID-19 cannot be ruled out as a possible reason. While the patient is currently at home and recovering, the cause of her hyponatremia is not clear. As there are few COVID-19 cases that exhibit hyponatremia, it is an area that needs further research, and in the interim, we advise clinicians to constantly look for electrolyte levels and pay close attention to medical management of COVID-19 patients.
